# Effect of GelMA Hydrogel Properties on Long-Term Encapsulation and Myogenic Differentiation of C_2_C_12_ Spheroids

**DOI:** 10.3390/gels9120925

**Published:** 2023-11-23

**Authors:** Karthika Muthuramalingam, Hyun Jong Lee

**Affiliations:** Department of Chemical and Biological Engineering, Gachon University, 1342 Seongnam-daero, Seongnam-si 13120, Gyeonggi-do, Republic of Korea; karthika2512@gachon.ac.kr

**Keywords:** C_2_C_12_ spheroids, skeletal muscle regeneration, GelMA, hydrogel-based scaffold

## Abstract

Skeletal muscle regeneration and engineering hold great promise for the treatment of various muscle-related pathologies and injuries. This research explores the use of gelatin methacrylate (GelMA) hydrogels as a critical component for encapsulating cellular spheroids in the context of muscle tissue engineering and regenerative applications. The preparation of GelMA hydrogels at various concentrations, ranging from 5% to 15%, was characterized and correlated with their mechanical stiffness. The storage modulus was quantified and correlated with GelMA concentration: 6.01 ± 1.02 Pa (5% GelMA), 75.78 ± 6.67 Pa (10% GelMA), and 134.69 ± 7.93 Pa (15% GelMA). In particular, the mechanical properties and swelling capacity of GelMA hydrogels were identified as key determinants affecting cell sprouting and migration from C_2_C_12_ spheroids. The controlled balance between these factors was found to significantly enhance the differentiation and functionality of the encapsulated spheroids. Our results highlight the critical role of GelMA hydrogels in orchestrating cellular dynamics and processes within a 3D microenvironment. The study demonstrates that these hydrogels provide a promising scaffold for the long-term encapsulation of spheroids while maintaining high biocompatibility. This research provides valuable insights into the design and use of GelMA hydrogels for improved muscle tissue engineering and regenerative applications, paving the way for innovative approaches to muscle tissue repair and regeneration.

## 1. Introduction

Skeletal muscle, the body’s most abundant and dynamic tissue, comprises approximately 40 percent of the total body mass in humans [1,2]. The high potential of the skeletal muscle’s self-repair and regeneration capacity majorly depends on a small population of adult stem cells called satellite cells [3,4]. While this regeneration capacity is limited only to small-scale muscle injuries, other conditions such as traumatizing injuries, degenerative muscle diseases, volumetric muscle loss, etc., cannot naturally restore muscle integrity, as the satellite cells therein are massively depleted [5]. Preserving muscle integrity by effectively guiding the restoration and regeneration of injured muscle tissues is critical for the normal physiological functioning of muscle tissues. Towards this, spatially organized cellular spheroids encapsulated in a 3D matrix play a significant role by mimicking elements of in vivo tissue architecture [6,7].

Cellular spheroids, three-dimensional dense cellular aggregates, mimic the complex in vivo microenvironment of cell–cell/cell–extracellular matrix interactions with physiological relevance under in vitro culture conditions [8,9]. They are considered in vitro building blocks of tissues, suitable for the bottom-up approach of tissue engineering and regenerative medicine (TERM) strategy. This in vitro 3D model is gaining much attention with its promising scientific validation when compared to flawed traditional 2D cell culture systems and time-consuming, expensive, and unreliable animal experimentations [10,11]. It serves as an effective tool for in vitro study models such as drug screening platforms, organ developmental studies, cell metabolism studies, and complex in vivo tissue regeneration constructs with structural complexity and homeostasis [12,13,14]. Scaffold-free cellular spheroids lack robust mechanical support, thereby deviating from maturing into desired functional tissue [15,16].

The controlled fabrication of matrix scaffolds with desired biocompatible and mechanical behaviors provides structural support and directs the encapsulated spheroids toward the regeneration of tissues of interest by staying at the site of implantation. Hydrogels, the crosslinked polymer network with the capability to absorb/retain relatively large amounts of water while retaining its 3D structures, are gaining significant attention towards spheroid encapsulation [17,18]. The characteristics of a hydrogel, such as a 3D network, possessing high water content, being highly porous, etc., that aid in the positive regulation of cell adhesion, cell migration, transportation of gasses/nutrients/wastes, etc., across the reticulated structure of hydrogels, thereby meeting the needs of encapsulated cells/spheroids, make hydrogels the desirable choice of scaffold for spheroid encapsulation. For better tissue regeneration, encapsulated spheroidal cells should migrate away from the spheroids, spread across the hydrogel scaffold, and reach out to the injured/defective tissue site, thereby resulting in an active tissue-like construct. Inside hydrogels, inter-spheroidal communication happens through paracrine signaling by spheroidal cell-secreted signaling molecules and/or a spheroidal cell communication bridge from cell sprouting.

Hydrogels should be fabricated with a mechanical and dynamic stiffness similar to that of native tissues to support the viability and functionality of cells/spheroids [19,20,21,22]. The more the compliance (i.e., lower stiffness) of the fibrinogen-modified poly(ethylene glycol)-diacrylate (PEG) hydrogel with a shear modulus of 2 kPa (~shear modulus of healthy liver tissue), the better the spheroid formation and growth of Huh7.5 cells compared with the stiffer PEG-based hydrogel [23]. Significant influence on podocyte behavior was observed when they were cultured in a hydrolyzed polyacrylamide (PAAm) hydrogel with an elasticity ranging from 0.9 to 9.9 kPa (aligning with the 2.5 kPa physiological stiffness of the kidney glomerular basement membrane) [24]. While a hyaluronic acid (HA) hydrogel with a storage modulus not exceeding 200 Pa influences the stemness of bone marrow mesenchymal stem cells (BMSCs), HA hydrogels with a mechanical strength greater than 1 kPa direct the chondrogenic differentiation of BMSCs [25]. When the stiffness of HA hydrogels increases from 28 to 250 Pa, the cultured induced neural stem cells (iNSCs) in the hydrogel alter their course from neural differentiation to glial cell differentiation, respectively, based on the varying elastic modulus [26]. Primary human chondrocytes cultured in fibrin hydrogel whose Young’s modulus represents that of perichondral space (~30 kPa) demonstrate better guidance for chondrocyte redifferentiation and synthesis of cartilage matrix components than in non-native physiological matrix stiffness of 1 to 15 kPa [27]. Thus, the mechanical properties of hydrogels have to be given key importance in scaffold fabrication for tissue engineering applications.

Yeo et.al. reported an in vitro scaffold model consisting of electro-spun C_2_C_12_ spheroid-laden alginate nanofibers on a microscale polycaprolactone (PCL) strut towards attaining a high degree of myoblast alignment and differentiation [28]. Though PCL is a biodegradable polymer, there exists a mechanical mismatch between the PCL strut reported therein and the native muscle tissue. Mechanical mismatches between engineered scaffold constructs and native tissue lead to reduced tissue output and implant failure [29,30,31]. Further, rather than a three-dimensional support system, the aligned nanofibrous bundle on the PCL strut provides a two-dimensional scaffolding support with topological cues. The impact of hydrogels on cellular behaviors such as cell sprouting, spreading, fusion, and differentiation in spheroid-based systems remains relatively obscure and requires extensive investigation.

In this study, we aimed to investigate the mechanical and biocompatible characteristics of a GelMA hydrogel with three different GelMA concentrations (5%, 10%, and 15%) on the functionality of C_2_C_12_ cellular spheroids formed with two different initial cell densities (1000 and 4000 cells/spheroid). Hydrogel-encapsulated spheroids were expected to exhibit enhanced cellular processes such as sprouting/spreading, migration, fusion, and differentiation. Our research aimed to elucidate the potential of GelMA hydrogel as a scaffold to promote cellular behaviors in the context of muscle tissue engineering and regenerative applications.

## 2. Results and Discussion

### 2.1. GelMA Hydrogel Fabrication and Characterization

**Scheme 1:**
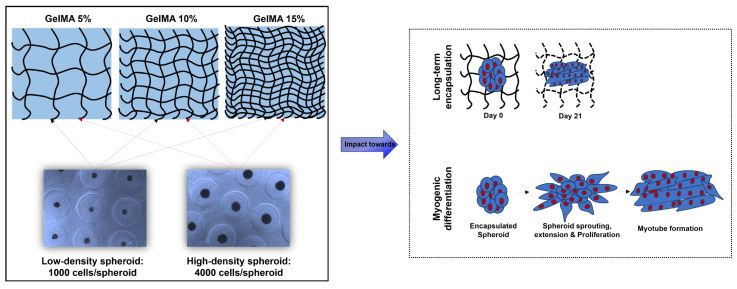
Schematic representation of GelMA hydrogels prepared with different concentrations, the formation of C_2_C_12_ myoblast spheroids with different cell densities, and the impact of a spheroid-encapsulated hydrogel towards long-term encapsulation and functionality of C_2_C_12_ spheroids.

**Figure 1:**
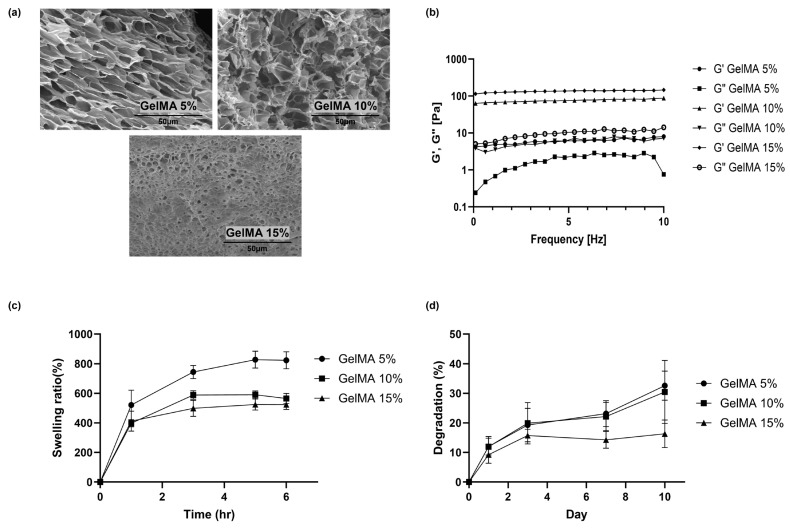
Mechanical and physical characterization of the GelMA hydrogel at different concentrations (5%, 10%, and 15%). (**a**) Cross-sectional scanning electron microscopy images of the lyophilized GelMA hydrogel; (**b**) rheological behavior (storage and loss moduli) of the GelMA hydrogel as a function of frequency; (**c**) swelling ratio; and (**d**) degradation of GelMA hydrogel.

Hydrogel preparation involved formulating GelMA at various concentrations ranging from 5% to 15%, effectively providing a wide range of mechanical properties to meet different cellular needs (Scheme 1). The selection of varying concentrations was driven by the need for the practical manageability of the formed hydrogels. A 5% GelMA hydrogel was chosen to maintain minimal feasibility for handling, while a 15% GelMA hydrogel was selected to ensure practical manageability. This practicality was experimentally demonstrated by assessing the viscoelastic characteristics of the differently concentrated hydrogels using rheometer measurements, as illustrated in Figure 1b. To facilitate hydrogel formation, the inclusion of Irgacure2959 as a photoinitiator was paramount, allowing the use of a standard photo-crosslinking technique. Subsequently, the application of UV radiation for only 20 s was sufficient to induce hydrogel formation. SEM images (Figure 1a) displayed a continuous and ordered porous scaffold structure of the lyophilized GelMA hydrogel with a homogenous architecture. The hydrogels were successfully formed by crosslinking the GelMA at concentrations ranging from 5 to 15%. In addition, the pore size of the hydrogel showed a remarkable reduction as the concentration of GelMA within the hydrogel matrix increased. The diameter of the porous structure corresponding to 5%, 10%, and 15% GelMA content were 19.1 ± 1.57, 14.8 ± 0.18, and 5.4 ± 1.8 μm, respectively. This indicated the correlation between GelMA content and pore size within the hydrogel matrix.

The mechanical properties of a substrate have been shown to have a profound effect on fundamental cellular processes, including adhesion, viability, proliferation, and differentiation. Using a frequency sweep experiment, as thoughtfully illustrated in Figure 1b, we delved into the rheological intricacies of the hydrogel. The conspicuous absence of a crossover frequency meant that the hydrogel collectives had undergone permanent crosslinking, rendering them intricately entangled over the entire range of the frequencies tested. Further, as the concentration of GelMA (5, 10, and 15%) increased, the storage modulus (6.01 ± 1.02, 75.78 ± 6.67, and 134.69 ± 7.93 Pa, respectively) increased, which indicated that the hydrogel with the highest GelMA content (15%) exhibited more elastic behavior and was the stiffest when comparing the other two study groups of 10% and 5%. It has been shown that the mechanical strength of the hydrogel can be easily adjusted by changing the concentration of GelMA. If mechanical strength is required under specific conditions, hydrogel materials can be prepared to meet these conditions.

Increasing hydrogel stiffness results in better adhesion, proliferation, and differentiation of primary mouse myoblasts in a standard 2D cell culture system [32]. C_2_C_12_ cells in a slowly degrading hydrogel system exhibit faster proliferation, while in faster degrading gels, differentiation is favored [32]. Stiffer HA/Gelatin hydrogels (with a Young’s modulus of 7.16 kPa) facilitated a higher density C_2_C_12_ myotube formation in the fabricated muscle cell sheet when compared with softer hydrogels with a Young’s modulus of 0.46 kPa and 3.98 kPa [33]. However, increasing the mechanical stiffness of collagen-alginate hydrogels impedes single-cell migration away from the spheroids, while reducing the mechanical stiffness permits migration into the hydrogel matrix [34]. The stiffer the collagen-agarose hydrogel, the more compact the spheroid, while the softest hydrogels result in cell protrusions and spatial dissemination [35]. Thus, the mechanical characteristics of the scaffold greatly impact the cellular events differently in 2D and 3D cell culture systems.

The transfer of nutrients, cellular metabolites, and ions under physiological conditions in and out of the hydrogel positively influence the maintenance and functionality of cultured cells and are highly associated with the swelling behavior of the hydrogel [36,37]. The degree of swelling (Figure 1c) was significantly higher in the 5% GelMA hydrogel than in the 10 and 15% GelMA hydrogel groups. A matrix with vulnerability to degradation plays a critical role in cellular proliferation, cellular migration, the metabolic activity of cells, cellular protrusions/outgrowth, and secretome composition, and thus the therapeutic potential of spheroids [38]. From Figure 1d, it is observed that GelMA hydrogels with 5 and 10% GelMA content showed a higher degree of degradation when compared with the 15% GelMA hydrogel.

### 2.2. C_2_C_12_ Spheroid Formation and Encapsulation in GelMA Hydrogels

The imprecise and uncontrolled differentiation of stem cells deteriorates the outcome of transplantation by failing to regenerate into a specific tissue of interest [39,40]. The unphysiological microenvironment of the single-cell suspensions raises concerns related to cell survival, cell retention, and the functionality of cells [10]. In addition, the loss of stemness and premature differentiation into multinucleated myotubes limit the use of myogenic stem cells or satellite cells in muscle tissue regeneration applications [41]. Supported by a scaffold for structural integrity, spheroids are favored over single-cell suspensions as they consume less time for skeletal muscle development, while in the latter case, myoblasts (the muscle progenitor cells) need to proliferate, differentiate, and then mature towards the formation of the myofiber [28]. However, in the case of spheroids, the increased cell-to-cell and cell-to-matrix interactions promote the rapid differentiation and maturation of myoblast cells. Hence, the suitability of GelMA-based hydrogel scaffolds for long-term encapsulation and better functionality of myogenic (C_2_C_12_) spheroids were demonstrated in this research.

**Figure 2:**
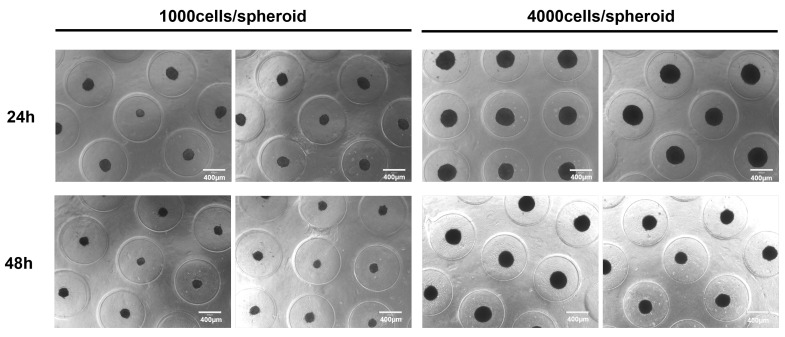
Optical images (scale bar: 400 μm) of C_2_C_12_ spheroid formation at 24 and 48 h with two different initial C_2_C_12_ cell seeding densities.

Not all cells can readily form a compact spheroid [42]. For the efficient transfer of gasses, nutrients, and wastes inside and outside of the spheroids, it is necessary to keep the spheroid size under control. Larger spheroids (>400 μm) develop necrotic cores with adjoining quiescent cell regions due to the depleted nutritional status arising from the hindered mass transportation across the spheroid [43]. Spheroid size is said to be affected by cell seeding density, cell type, and culture time. The ability of C_2_C_12_ cells towards spheroid formations from two different cell seeding densities (1000 and 4000 cells per spheroid) was microscopically observed and is shown in Figure 2. C_2_C_12_ spheroids as loosened cell aggregates were formed within the initial 24 h of cell seeding. Compact spheroid formation was observed at the end of 48 h after the initial seeding. The diameter of the spheroids was reduced from 219.34 ± 23.89 and 357.02 ± 20.33 μm at 24 h to 167.18 ± 14.08 and 285.51 ± 25.45 μm at 48 h in the groups of 1000 cells/spheroid and 4000 cells/spheroid, respectively. Thus, the 48 h old spheroid was further taken for encapsulation into the GelMA hydrogel.

**Figure 3:**
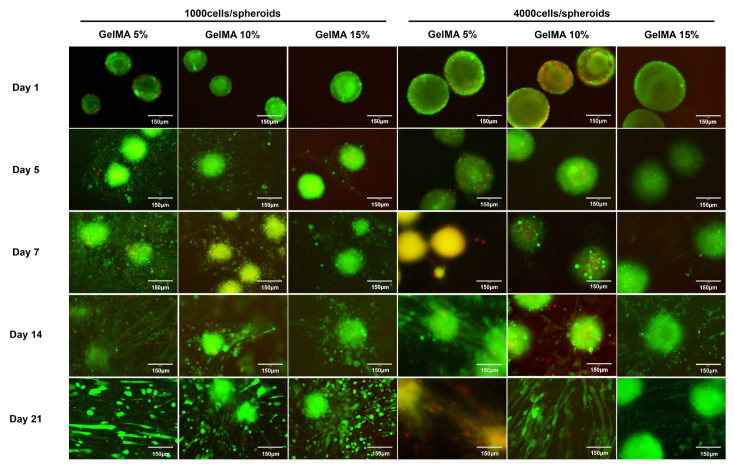
Live (green)/Dead (red) fluorescent images (magnification: 20×; scale bar: 150 μm) of encapsulated C_2_C_12_ spheroids in GelMA hydrogel (5%, 10%, and 15%) at different time points.

Images of Live/Dead staining (a qualitative viability testing) of C_2_C_12_ spheroids encapsulated in 5%, 10%, and 15% GelMA hydrogels at different time scales are shown in Figure 3. UV irradiation for hydrogel crosslinking appeared minimally toxic and did not affect the viability of the cells in the spheroids in any of the study groups. It was observed that the GelMA hydrogel held the encapsulated spheroids with better integrity and functionality, even on day 21. The spreading of spheroids resulted in the breakage of their initial circular shape. Cell sprouting and migration occurred faster (as shown on day 5) in the low-percentage (5%) GelMA hydrogel with a low-density (1000 cells/spheroid) spheroid than in the rest of the groups. The large surface area (provided by the high-porous structure) and greater swelling ability of the 5% GelMA hydrogel supported the enhanced cell sprouting and migration of spheroidal cells. Sprouting and migration were minimal even on day 7 in the high-density spheroid-encapsulated GelMA hydrogel groups, regardless of the different concentrations of the GelMA hydrogel. High-density spheroids and high GelMA concentrations in the hydrogel were found to impact the cell sprouting and migration away from the spheroid, thus affecting the functionality of the spheroid. Enhanced cell spreading resulted in increased cell-to-cell contact around the spheroids, which was required for the induction of myotube formation. The spatial alignment of component cells is one hallmark of muscle tissue, wherein the densely packed muscle cells in 3D structures, when amenable to align with one another in a preferential direction, result in a successfully bioengineered muscle construct. Seyedmahmoud et.al. fabricated 3D bioprinted muscle tissue constructs using a C_2_C_12_ cell-laden GelMA-alginate hydrogel, wherein they observed that increased cell-spreading, cell–cell interaction, and cell–cell fusion offered by the GelMA-alginate bioink supported the myoblast differentiation process [44]. Demri et.al. proposed a multiscale magnetic approach for anisotropic tissue engineering with a magnetic nanoparticle-loaded muscle cell model, where they observed multinucleated cells within fibers formed from the fusion of spheroids into 3D tubular structures in a thermoresponsive collagen hydrogel, oriented in the direction of magnetic alignment [45]. Thus, the cellular events of sprouting/migration, proliferation, and differentiation result in the successful outcome of tissue constructs with physiological functionality.

### 2.3. Myogenic Differentiation of C_2_C_12_ Spheroid in GelMA Hydrogels

**Figure 4:**
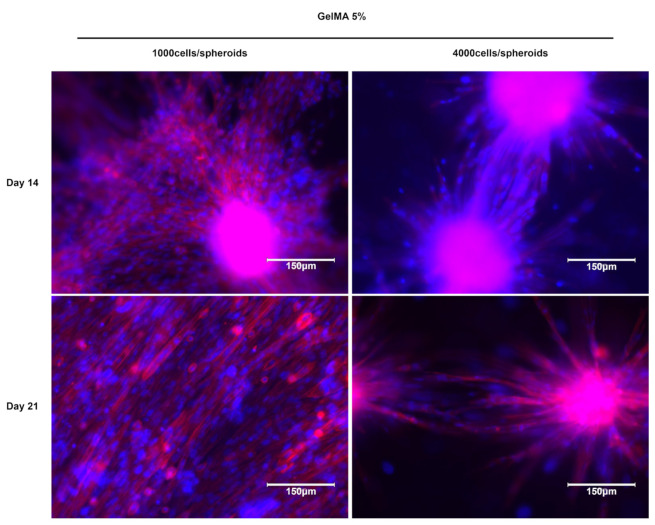
DAPI (blue)/phalloidin (red) fluorescent images (magnification: 20×; scale bar: 150 μm) of C_2_C_12_ spheroids encapsulated in a 5% GelMA hydrogel at day 14 and 21.

Myotube formation was clearly observed in the 5% GelMA hydrogel, and this phenomenon was more pronounced in the low-density spheroids at the 14-day mark. Notably, this myotube formation was accompanied by the establishment of spheroid-to-spheroid communication. Myotube shape and structure were visualized using phalloidin staining, which highlighted force-generating filamentous actin, and DAPI staining was used for the nuclei. This visualization is shown in Figure 4.

The early sprouting and migration of cells away from the low-density spheroids within the 5% GelMA hydrogel likely facilitated the ability of myoblasts to proliferate, align, fuse, and differentiate into myotubes earlier than in the high-density spheroid-encapsulated 5% GelMA hydrogel group. As a result, the actin filaments were significantly thicker and showed elongation in the low-density spheroid-encapsulated 5% GelMA hydrogel. This observation underscored the critical role of cell density and hydrogel concentration in influencing the dynamics of myotube formation, with low-density spheroids and 5% GelMA hydrogels providing an environment conducive to early and robust myotube development as evidenced by the structural characteristics of actin filaments.

**Figure 5:**
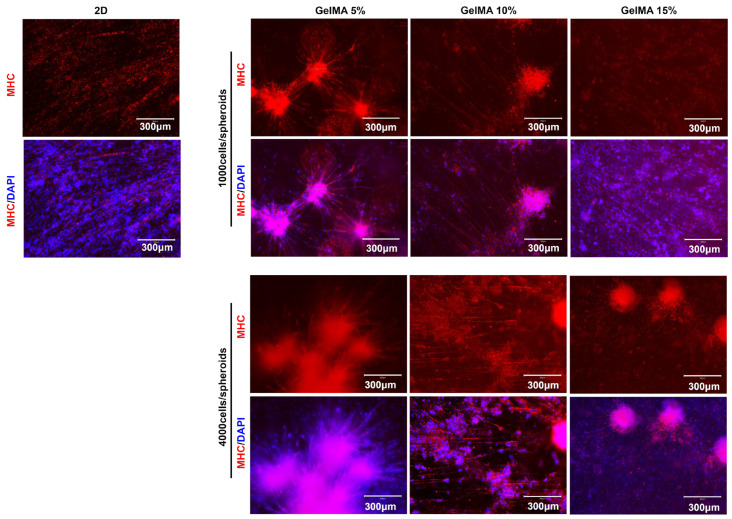
MHC immunofluorescence staining images (DAPI (blue)/MHC (red); magnification: 10×; scale bar: 300 μm) of C_2_C_12_ spheroids encapsulated in the GelMA hydrogel (5%, 10%, and 15%) at day 21.

The results of myosin-heavy-chain (MHC) staining, a well-established marker for myotube formation, provided insights into the development of multi-nucleated myotubes arising from differentiated myoblast cells (see Figure 5). Importantly, the level of myotube maturity, as determined by the intensity of MHC expression relative to the imaging area of the hydrogel, was notably higher in the 5% and 10% GelMA hydrogels. Remarkably, this held true for both the low and high spheroid densities.

This finding suggested that the 5% and 10% GelMA hydrogels created an environment that was particularly conducive to myotube maturation, irrespective of the initial spheroid density. The robust MHC expression and the prevalence of multi-nucleated myotubes in these hydrogels underscored their potential to effectively support myoblast differentiation and myotube formation. The intensity of MHC expression served as a strong indicator of the advanced stage of myotube development, highlighting the suitability of these hydrogel formulations for promoting muscle tissue maturation.

Taken together, our observations revealed a critical interplay between the mechanical properties and swelling capacity of the GelMA hydrogel, with profound consequences for the dynamics of cell sprouting and migration from the C_2_C_12_ spheroids. This intricate relationship, in turn, served as a catalyst for enhancing the differentiation and overall functionality of C_2_C_12_ spheroids. Furthermore, our results supported the remarkable ability of the GelMA hydrogel to serve as an efficient encapsulation matrix for C_2_C_12_ spheroids, ensuring their long-term retention while maintaining exceptional biocompatibility. This multifaceted relationship between substrate properties, cellular dynamics, and biocompatibility is critical to advancing our understanding of tissue engineering and regenerative applications and offers promising insights for future efforts in this field.

## 3. Conclusions

This study highlighted the pivotal role of gelatin methacrylate (GelMA) hydrogels in the context of cellular spheroid encapsulation and its implications for muscle tissue engineering and regenerative applications. The ability to fabricate GelMA hydrogels with stable structural integrity and varying mechanical properties was demonstrated, allowing for a comprehensive study of their impact on cellular behavior. The mechanical characteristics of GelMA hydrogels, spanning concentrations from 5% to 15%, were observed to exert a significant impact on cell adhesion, survival, proliferation, and differentiation. GelMA hydrogels with a heightened swelling capability facilitated cell sprouting and migration away from C_2_C_12_ spheroids, thereby enhancing spheroidal functionality, while GelMA hydrogels with elevated stiffness constrained these cellular events. In addition, the long-term encapsulation and biocompatibility of C_2_C_12_ spheroids within GelMA hydrogels were effectively demonstrated. The mechanistic insights provided in this study elucidate how GelMA hydrogel properties could be tailored to promote the functionality of encapsulated spheroids, thereby succeeding in tissue engineering and regenerative applications.

## 4. Materials and Methods

### 4.1. Materials

Gelatin Methacrylate (GelMA) with a degree of methacrylation ≥ 90% was purchased from 3D Materials, Daejeon, Republic of Korea. Irgacure2959 (2-Hydroxy-4′-(2-hydroxyethoxy)-2-methylpropiophenone), Bovine Serum Albumin (A7906, purity ≥ 98%), Triton™ X-100 (laboratory grade), and paraformaldehyde were purchased from Sigma Aldrich (St. Louis, MO, USA). Cellove™ Dulbecco’s Phosphate-Buffered Saline (DPBS) and Dulbecco’s Modified Eagle’s Medium (high glucose) (DMEM) were purchased from SolBio, Gyeonggi-do, Republic of Korea. Fetal Bovine Serum (FBS) (Gibco, Grand Island, NY, USA), Horse Serum (HS) (Cytiva, Marlborough, MA, USA), and Penicillin/Streptomycin (P/S) 100× (Welgene, Daegu, Republic of Korea) were purchased. Agar Powder (extra-pure grade) was purchased from Duksan Reagents, Gyeonggi-do, Republic of Korea. Gibco™ Phosphate-Buffered Saline (PBS, pH 7.4 (1×)), Invitrogen™ Rhodamine Red™—X goat anti-mouse IgG (H + L), and an Invitrogen™ Live/Dead™ viability/cytotoxicity kit was obtained from Thermo Fischer Scientific Korea Co., Ltd., Seoul, Republic of Korea. An F-actin staining kit—red fluorescence—Cytopainter (ab112127) was obtained from Abcam (Cambridge, UK). Myosin Heavy Chain 2 (sc-53095) antibodies were obtained from Santa Cruz Biotechnology, Inc (Dallas, TX, USA).

### 4.2. GelMA Hydrogel Fabrication

GelMA at three different concentrations (5%, 10%, and 15% *w*/*v*) was prepared by dissolving the appropriate amount of GelMA powder in PBS (80% of the desired final volume) under heat at 70 °C until it dissolved. By placing it in a 70 °C water bath, dissolve Irgacure2959 in PBS, which consists of the remaining 20% of the desired final volume, so that its final concentration in the GelMA-Irgacure2959 solution is 0.5% *w*/*v*. The Irgacure2959 precursor solution should be protected from light at all steps until hydrogel crosslinking is initiated. Mix the precursor solutions of GelMA and Irgacure2959 for 10 min under heat at 40 °C. Photoinitiator-added GelMA solution was placed under a UV light source (Altis UVcure, Incheon, Republic of Korea—Spot UV LED Curing System—365 nm, 5980 mW/cm^2^) and was irradiated for 20 s to initiate crosslinking. The UV light source should be evenly exposed across the sample at a constant sample-UV source gap.

### 4.3. GelMA Hydrogel Characterization

The morphology of the hydrogel samples was characterized using scanning electron microscopy (SEM) (SU8600, Hitachi, Tokyo, Japan) with an accelerating voltage of 5 kV under vacuum conditions at the Smart Materials Research Center for IoT, Gachon University, Korea. The prepared hydrogel was subjected to lyophilization for 24 h before SEM characterization. A vertical cross-sectional cut was made on the lyophilized sample, gold-coated to prevent charge accumulation on the sample surface, mounted on a stub using sticky carbon tape, and proceeded to SEM analysis. The rheological behavior analysis of the hydrogel was performed using an MCR92 Modular Compact Rheometer (Anton Paar, Graz, Austria) in a parallel-plate geometry cell with a gap between plates of 0.5 mm. A total of 100 μL of hydrogel precursor solution was placed on the plate, and crosslinking was initiated by exposing it to UV light for 20 s, followed by a measurement of storage (G’) and loss (G”) moduli in the frequency range of 0.1 to 10 Hz at room temperature. To determine the degree of swelling of the lyophilized hydrogel, they were immersed in a predetermined volume of PBS at 37 °C. At the indicated time, the swollen hydrogel was weighed after removing excess water by gently blotting using tissue paper. The experiment continued until no difference in the swelling ratio was observed. The swelling percentage was calculated from the following equation: degree of swelling (%) = (W_s_ − W_d_/W_d_) × 100, where W_s_ and W_d_ are the weights of the hydrogel sample in the swollen and lyophilized states, respectively. Quantitative values are expressed as means ± standard deviation; sample size, n = 3.

As-prepared hydrogel samples were immersed in PBS at 37 °C. The weight of the hydrogel was measured before and after immersion at the indicated time point. Fresh PBS was replaced every 48 h. In vitro degradation was calculated as follows: degradation (%) = (W_0_ − W_t_/W_0_) × 100, where W_0_ and W_t_ are the weights of the as-prepared hydrogel and weight of the hydrogel at the indicated time point, respectively. Quantitative values are expressed as means ± standard deviation; sample size, n = 3.

### 4.4. Cell Culture

Mouse myoblast cell line C_2_C_12_ (CRL-1772™, ATCC, Manassas, VA, USA) was cultured in DMEM (high glucose) containing 10% FBS and 1% P/S at 37 °C in a 5% CO_2_ condition. For the differentiation studies, the growth medium was switched to a differentiation medium containing DMEM (high glucose) with 2% HS and 1% P/S at 37 °C in 5% CO_2_. Cells under passage 12 were used for spheroid formation.

### 4.5. Spheroid Formation and Encapsulation

C_2_C_12_ spheroids were fabricated in 3D Petri dishes templated using micro-molds (MicroTissues Inc., Providence, RI, USA), following the manufacturer’s protocol. Briefly, molten agar solution was poured onto the 24-series micro-mold, allowed to gel, and equilibrated with a cell culture medium. After removing the medium, cell suspension in the growth medium was seeded and allowed to settle for ~15 min into the features of the 3D Petri dish. Following 48 h of cell aggregation into spheroids, the spheroids were harvested and mixed with a GelMA hydrogel precursor solution. Crosslinking was initiated by exposing the GelMA hydrogel precursor solution/spheroid mixture under UV light for 20 s. The spheroid-encapsulated hydrogel was kept in a differentiation medium, and spheroid viability, differentiation, and protein expression were observed at different time points.

### 4.6. Characterization of Spheroids Encapsulated within Hydrogels

The viability of spheroids encapsulated within hydrogels was assessed with a Live/Dead assay. Briefly, the spheroid-laden hydrogel was incubated in a calcein-AM/ethidium homodimer-1 solution (dissolved in DPBS) for 30 min, and then observed with an EVOS M5000 Imaging System (Thermo Fischer Scientific). For immunocytochemical analysis, the spheroid-laden hydrogel was washed with PBS and fixed in 4% formaldehyde for 30 min. Following fixation, permeabilization was performed with 0.1% Triton X100 in PBS for 10 min. After washing, the blocking solution (3% BSA prepared in PBS) was added and incubated for 1 h at room temperature. The primary antibody at the desired concentration was then added and incubated overnight at 4 °C. PBS washing was performed after removing the primary antibody solution, followed by secondary antibody incubation at room temperature in the dark for 2 h. A nucleus staining solution was added after washing with PBS and incubating in the dark for 10 min at room temperature. The sample was then taken for fluorescent imaging. Filamentous actin (F-actin) was stained with a red fluorescent phalloidin conjugate solution. For F-actin staining, after the permeabilization and PBS washing of the spheroid-laden hydrogel, F-actin solution was added and incubation was performed for 1 h, followed by nucleus staining for 10 min and imaging.

## Data Availability

Data are contained within the article.
